# Depression Disorder Classification of fMRI Data Using Sparse Low-Rank Functional Brain Network and Graph-Based Features

**DOI:** 10.1155/2017/3609821

**Published:** 2017-04-12

**Authors:** Xin Wang, Yanshuang Ren, Wensheng Zhang

**Affiliations:** ^1^Institute of Automation, Chinese Academy of Sciences, Beijing 100190, China; ^2^Department of Radiology, Guang'anmen Hospital, China Academy of Chinese Medical Sciences, Beijing 100053, China

## Abstract

Study of functional brain network (FBN) based on functional magnetic resonance imaging (fMRI) has proved successful in depression disorder classification. One popular approach to construct FBN is Pearson correlation. However, it only captures pairwise relationship between brain regions, while it ignores the influence of other brain regions. Another common issue existing in many depression disorder classification methods is applying only single local feature extracted from constructed FBN. To address these issues, we develop a new method to classify fMRI data of patients with depression and healthy controls. First, we construct the FBN using a sparse low-rank model, which considers the relationship between two brain regions given all the other brain regions. Moreover, it can automatically remove weak relationship and retain the modular structure of FBN. Secondly, FBN are effectively measured by eight graph-based features from different aspects. Tested on fMRI data of 31 patients with depression and 29 healthy controls, our method achieves 95% accuracy, 96.77% sensitivity, and 93.10% specificity, which outperforms the Pearson correlation FBN and sparse FBN. In addition, the combination of graph-based features in our method further improves classification performance. Moreover, we explore the discriminative brain regions that contribute to depression disorder classification, which can help understand the pathogenesis of depression disorder.

## 1. Introduction

As one of the most prevalent psychiatric disorders, depression disorder is typically characterized by persistent depressed mood, loss of motivation, and sleep abnormalities [1]. Moreover, it can lead to suicide at its worst. According to the World Health Organization, an estimated 350 million people of all ages suffer from depression disorder globally [2]. However, the diagnosis of depression disorder mainly depends on clinical symptoms, and its pathogenesis remains unclear [3]. Functional magnetic resonance imaging (fMRI) can contribute to the diagnosis and a better understanding of the pathogenesis of depression disorder [4, 5]. This brain imaging technique provides an effective tool to explore functional abnormalities of depression disorder [6].

A large number of fMRI studies have reported abnormal functional brain network (FBN) in patients with depression [7, 8]. However, the models to construct FBN suffer from several limitations. FBN is a mathematical representation of brain. Brain regions are nodes and functional connectivities between each pair of brain regions are edges. Pearson correlation is the most commonly used model for constructing FBN, in which the functional connectivity (FC) value is estimated by the correlation coefficient between brain regions [9]. Connolly et al. use Pearson correlation to analyze the abnormal FC between subgenual anterior cingulate cortex and other brain regions in depressed adolescents [10]. However, it only captures pairwise information between brain regions without considering influence of other brain regions. Independent Component Analysis (ICA) can also be used to construct FBN by grouping brain regions into latent components. The brain regions within the same component are believed to have strong FC, while the FC between different components is weak [11, 12]. Increased FC between subgenual cingulate and thalamic is detected in patients with depression by ICA [13]. The main drawbacks of ICA are the inaccessibility of FC value and the uninterpretability of components. Recent work tries to impose sparse prior to the models for constructing FBN. It is based on neurological findings that a brain region usually only directly interacts with a few other brain regions [14]. Huang et al. construct FBN by employing a sparsity prior in the estimation of inverse covariance matrix [11]. Although this sparse representation model calculates FC between each pair of brain regions with consideration of all the other brain regions, the sparsity prior is not enough to describe the structure of FBN.

As the functional abnormalities of depression can be explored by FBN, many classification methods based on FBN are developed for depression disorder classification. Feature extraction plays a key role in the classification methods. FC in FBN can be directly used as a feature for depression disorder classification [15, 16]. Zeng et al. use multivariate pattern analysis to classify FC of patients with depression and FC of healthy controls. In addition, regional homogeneity and amplitude of low frequency fluctuations are also commonly used features for depression disorder classification [17, 18]. However, these features, which only consider the specific local changes of FBN, are not effective for classification. A more comprehensive feature extraction approach is needed for depression disorder classification.

To overcome the limitations lying in construction of FBN and feature extraction, we propose a new method for depression disorder classification. In this paper, FBN is constructed by sparse low-rank model and eight graph-based features are extracted for classification. Sparse low-rank model provides a much better FBN than Pearson correlation or simple sparse representation model for three reasons. First, FBN constructed by sparse low-rank model considers the linear relationship between two brain regions given all the other brain regions, in contrast with the pairwise Pearson correlation. Secondly, imposing sparsity on FBN is interpretable because a brain region only directly interacts with a few other brain regions in neurological processes, which has been supported by some neurophysiological findings [14, 19]. Thirdly, low-rank constraint encodes a modular structure to the FBN, which is closer to the real FBN [20, 21]. Sparse representation and dictionary learning can also be used as a classifier for fMRI data. Our previous work proposes a weighted discriminative dictionary learning (WDDL) method for disease classification [22]. The model of WDDL represents each test sample using two class-specific dictionaries, respectively, and classifies it to the class with the smaller representation error. However, in this work, we detect the effect of a sparse low-rank model to construct FBN, which is a part of feature extraction for classification.

Once the FBN is constructed by sparse low-rank model, we extract eight graph-based features, which provide information about the entire network other than specific local changes [23, 24]. The eight graph-based features are from the aspects of functional segregation, functional integration, nodal centrality, and network resilience. We choose graph-based features to measure FBN for two reasons. First, graph-based features are effective in helping us understand the functional organization of network and ranging from cells [25] and tissues [26, 27] to the whole ecosystems [28, 29]. Secondly, recent researches have shown that graph-based features, which measure topological properties of FBN, make the classification methods have good classification performance [30, 31].

**Figure 1:**
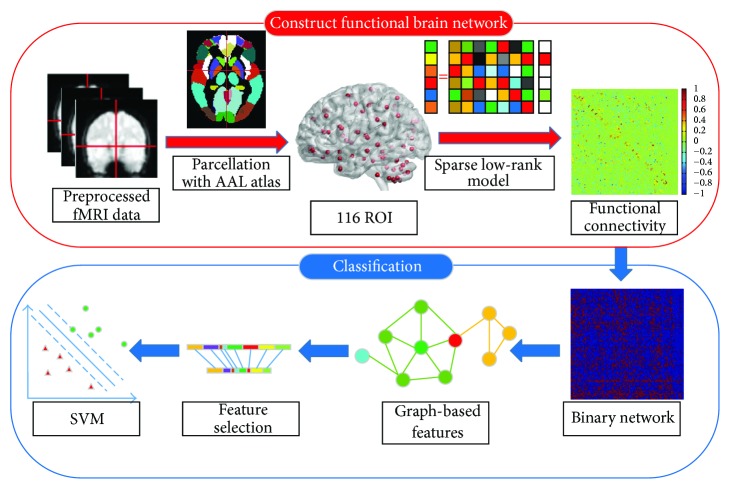
The schematic diagram of our method for depression disorder classification. SVM: support vector machine.

In short, the main contributions of this paper are as follows: (1) FBN is constructed by sparse low-rank model, which can calculate the relationship between two brain regions given all the other brain regions. (2) We extract eight graph-based features, which can effectively characterize the FBN from different aspects. To our knowledge, this is the first study of depression disorder classification, which extracts graph-based features from sparse low-rank FBN. The experimental results show that both sparse low-rank FBN and the combination of graph-based features improve the classification performance. Generally, the promising classification result proves the effectiveness of our method. The overall procedure of our method is shown in Figure 1.

## 2. Methods

### 2.1. Participants, Data Acquisition, and Preprocessing

31 patients with depression (16 females, 15 males) and 29 age-, sex-, and education-matched healthy controls (15 females, 14 males) are recruited from the Department of Radiology, Guang'anmen Hospital of China Academy of Traditional Chinese Medicine. The average age of patient group and control group is 30.42 and 32.63, respectively. All subjects are right-handed native Chinese speakers. Written informed consent is obtained from all subjects. All the patients with depression are diagnosed according to Structured Clinical Interview for the DSM-IV, patient version (SCIDI/P) [32], by experienced psychiatrists. They have no history of other neurological illness or head injury. Healthy controls are interviewed using the Structured Clinical Interview for DSM-IV, nonpatient edition (SCIDI/NP). They have no current or history of depression disorder or other psychiatric disorders.

The fMRI measurements are performed on a General Electric (GE) signa 1.5T echo speed superconducting MRI scanner. Functional images are acquired with an echo-planar imaging (EPI) sequence: repetition time (TR) = 2000 ms, echo time (TE) = 30 ms, flip angle = 90°, field of view (FOV) = 24 cm, matrix = 64 × 64, thickness = 3 mm, and slices = 41. Subjects are instructed not to think of anything and keep their eyes closed but not fall asleep during the resting-state fMRI acquisition. For each subject, the fMRI scanning lasts for four minutes and twenty-eight seconds and 144 volumes are obtained.

The preprocessing of fMRI data is conducted using Statistical Parametric Mapping (SPM8, http://www.fil.ion.ucl.ac.uk/spm/software/spm8/), Resting-State fMRI Data Analysis Toolkit (REST, http://restfmri.net/forum/index.php), and Data Processing Assistant for Resting-State fMRI (DPARSF, http://www.restfmri.net/forum/taxonomy/term/36). The first 10 time points are discarded for subject's adaptation to the scanning and the scanner calibration. The remaining images are first corrected for different slice acquisition timing and head motion. No subject is discarded for excessive head movement (translation < 2.0 mm or rotation < 2.0°). Next, the images are spatially normalized to the standard EPI template in SPM8 and resampled to a voxel size of 3 × 3 × 3 mm^3^. After this, the images are smoothed with an isotropic Gaussian kernel (FWHW = 4 mm) and temporal band-pass filtered (0.01 Hz–0.08 Hz). To further reduce the effects of nuisance signals, regression of 6 head motion parameters, global mean signal, white matter signal, and cerebrospinal fluid signal are performed. Finally, we use the Automated Anatomical Labeling (AAL) atlas [33] to segment brain signals. The mean fMRI time series of 116 brain regions are obtained for further analysis. After preprocessing, the final number of volumes is 134 as 10 volumes are discarded from the 144 volumes. The dimensionality of data matrix is 134*∗*116 for each subject.

### 2.2. Construction of FBN

FBN is a mathematical representation of the system of brain, which is defined by a collection of nodes and edges [24, 34]. In this paper, nodes represent the brain regions obtained from AAL atlas. Edges linking two nodes represent the FC between the two corresponding brain regions. FC is defined as statistical dependency between spatially remote brain regions [35, 36]. A high correlation between the time series of the two brain regions reflects a high level of FC between them.

FBN has many inherent structures, some of which can guide to construct a better FBN. Sparsity and modularity are two important structures of FBN, which can be used by adding some constraints to the constructed model. Sparsity means that a brain region only directly interacts with a few other brain regions in neurological processes [14, 19]. The sparsity prior can be used in FBN construction by adding *ℓ*_0_-norm or *ℓ*_1_-norm constraint to the objective function. In addition, modularity refers to that there exist some node groups (communities) in the FBN [24]. The FC between nodes from the same group is dense, while FC between nodes from different groups is sparse. It has proved that the combining of sparse and low-rank constraint can describe the modularity of FBN [21]. Therefore, we use a sparse low-rank model to construct FBN in this paper. The reasons for choosing sparse low-rank model for FBN construction are as follows: (1) the sparse low-rank model can construct FBN with both sparse and modular structure, which is verified in Results. (2) The classification performance can be improved by sparse low-rank model, compared with the commonly used Pearson coefficient model and sparse representation model, as shown in Results.

The sparse low-rank model can be used to construct FBN as follows. Assuming we have *N* subjects, each of which has *m* brain regions. Let **X** = [**x**_1_,…, **x**_**m**_] ∈ *ℝ*^*t*×*m*^ be the fMRI data matrix of a subject, where *t* is the number of time points. For the time series of each brain region **x**_**i**_, we use the time series of all the other brain regions **X**_**i**_ = [**x**_1_,…, **x**_**i**−1_, **x**_**i**+1_,…, **x**_**m**_] ∈ *ℝ*^*t*×(*m*−1)^ as dictionary to represent this brain region with coding coefficient **a**_**i**_, namely, **x**_**i**_ = **X**_**i**_ × **a**_**i**_.

The sparse low-rank FBN of the *n*th subject can be formulated as the following objective function:

(1) JA=arg minA⁡X−XAF2+λ1A0+λ2rankA,

where **A** = [**a**_1_, **a**_2_,…, **a**_**m**_] is the coding coefficient matrix. The *j*th element of **a**_**i**_ denotes the relationship between **x**_**i**_ and **x**_**j**_ given all the other **x** in **X**_**i**_. Then, the matrix **A** is a FC matrix of subject **X**. And the FC between two brain regions are calculated given all the other brain regions, compared with the pairwise Pearson correlation. This is also a reason that we choose sparse low-rank model to construct FBN. *λ*_1_ and *λ*_2_ are the regularization parameters for trade-off among the three terms. The first term is the data-fitting term, the second term is sparsity constraint, and the last term is low-rank constraint on the FC matrix **A**. With the introduction of those two constraint terms, the constructed FBN is imposed to have sparse and modular structure. As the two constraint terms are both nonconvex with respect to **A**, they are relaxed to *ℓ*_1_-norm ‖**A**‖_1_ and trace norm ‖**A**‖_*∗*_, respectively. The objective function in (1) can be written as follows:

(2) $$\begin{array}{c} J\left(\;A\;\right)=arg\;\underset{A}{\min}\left(\;{\left\|\;X-XA\;\right\|}_{F}^{2}+\lambda_{1}{\left\|\;A\;\right\|}_{1}+\lambda_{2}{\left\|\;A\;\right\|}_{*}\;\right), \end{array}$$

where ‖**A**‖_1_ = ∑_*i*_∑_*j*_|**a**_*ij*_|. The objective function can be optimized via a proximal method [37]. Once the optimal FC matrix **A** is obtained, we replace **A** with $\tilde{A}=(A+A^{T})/2$ to obtain a symmetry FC matrix. The replacement is based on a discovery that asymmetry of the FC matrix does not contribute to the final classification performance [21]. In addition, all the diagonal elements of the FC matrix (self-connections) are set to zero.

### 2.3. Feature Extraction

To extract effective graph-based features from the constructed FBN, the original FC matrices are first converted to binary matrices by setting all the nonzero connectivity to one. In this paper, eight graph-based features are computed from the following four aspects: functional segregation, functional integration, nodal centrality, and network resilience [24].

#### 2.3.1. Functional Segregation

Functional segregation measures how efficiently information is exchanged within interconnected groups of brain regions.

Clustering coefficient is defined as the number of neighbors of a given node connected to its other neighbors, which describes the level of local neighborhood clustering of a network [38]. The clustering coefficient of node *i* is defined as

(3) $$\begin{array}{c} C_{i}=\frac{2r_{i}}{k_{i}\left(k_{i}-1\right)}, \end{array}$$

where *r*_*i*_ is the number of triangles around a node *i* and *k*_*i*_ is the degree of node *i* which will be described below.

Local efficiency describes how efficient is the communication between the first neighbors of node *i* when the node is removed [39]. The local efficiency is the average of inverse shortest path length between the direct neighbors of a node. It is defined as

(4) $$\begin{array}{c} E_{loc,i}=\frac{\sum_{j,h\in G_{i}}{\left[d_{jh}\left(G_{i}\right)\right]}^{-1}}{k_{i}\left(k_{i}-1\right)}, \end{array}$$

where *G*_*i*_ is the set of nodes that are neighbors of node *i* and *d*_*jh*_(*G*_*i*_) is the shortest path length between node *j* and node *h*, which contains only direct neighbors of node *i*.

#### 2.3.2. Functional Integration

Functional integration is used to measure the ability of brain to rapidly integrate information from distributed brain regions. Characteristic path length [40] and global efficiency [39] are the two most commonly used measures of functional integration. The global efficiency is the average inverse shortest path length. They are respectively defined as

(5) L=1n∑i∈N∑j∈N,j≠idijn−1,E=1n∑i∈N∑j∈N,j≠idij−1n−1,

where *L* and *E* are the characteristic path length and global efficiency of the network, *n* is the number of nodes in the network, *N* is the set of all the nodes in the network, and *d*_*ij*_ is the shortest path length between node *i* and node *j*.

#### 2.3.3. Nodal Centrality

Degree and betweenness centrality are used to measure the centrality of a node. Degree of a node is defined as the number of links connected to the node, which reflect the importance of a node. Degree of node *i* is defined as

(6) $$\begin{array}{c} k_{i}=\underset{j\in N}{\sum }g_{ij}, \end{array}$$

where *g*_*ij*_ is the connection status between node *i* and node *j*: *g*_*ij*_ = 1 when link (*i*, *j*) exists and *g*_*ij*_ = 0 otherwise.

Betweenness centrality of a node is defined as the fraction of all shortest paths that pass through the node [41]:

(7) $$\begin{array}{c} b_{i}=\frac{1}{\left(n-1\right)\left(n-2\right)}\underset{\begin{array}{c} \overset{h,j\in N}{h\neq j,h\neq i,j\neq i} \end{array}}{\sum }\frac{\rho_{hj}\left(i\right)}{\rho_{hj}}, \end{array}$$

where *ρ*_*hj*_(*i*) is the number of shortest paths between node *h* and node *j* that pass through node *i* and *ρ*_*hj*_ is the number of all the shortest paths between node *h* and node *j*.

Participation coefficient assesses the diversity of intermodular interconnections of individual nodes. The participation coefficient of node *i* is defined as

(8) $$\begin{array}{c} y_{i}=1-\underset{m\in M}{\sum }{\left(\;\frac{k_{i}\left(m\right)}{k_{i}}\;\right)}^{2}, \end{array}$$

where *M* is the set of modules and *k*_*i*_(*m*) is the number of links between *i* and all nodes in module *m*.

#### 2.3.4. Network Resilience

Indirect measures of resilience quantify anatomical features that reflect network vulnerability to insult. Among these measures, a typical one is average neighbor degree [42]:

(9) $$\begin{array}{c} k_{nn,i}=\frac{\sum_{j\in N}g_{ij}k_{j}}{k_{i}}. \end{array}$$

Once we have obtained all the eight graph-based features, we concatenate them to construct the final feature vectors. Specifically, for each subject, the feature vector has a size of 698, which consists of 116*∗*6 local measures and 2 global ones. The dimensionality of feature matrix is 698*∗*60, which consists of the feature vectors of all the subjects. As leave-one-out cross-validation (LOOCV) is used for classification, the training matrix dimensionality is 698*∗*59 in each LOOCV.

### 2.4. Feature Selection

The goal of feature selection is to remove irrelevant or redundant features and retain discriminative features, which can lead to a better classification performance of the model. In this paper, we employ Fisher score to select useful features. Fisher score is used to describe the discriminatory power of a feature between two classes [30, 43]. Fisher score for each feature is defined as

(10) $$\begin{array}{c} FS=\frac{p_{1}{\left(q_{1}-q\right)}^{2}+p_{2}{\left(q_{2}-q\right)}^{2}}{p_{1}\sigma_{1}^{2}+p_{2}\sigma_{2}^{2}}, \end{array}$$

where *p*_1_ and *p*_2_ are the numbers of samples in the two classes, *q*_1_ and *σ*_1_^2^ are the feature mean value and variance of one class, *q*_2_ and *σ*_2_^2^ are the feature mean value and variance of the other class, and *q* is the feature mean value of all the samples.

A larger Fisher score indicates a more discriminative feature. We rank all the features in the training set based on Fisher score. Different feature sets can be obtained by selecting different number of ordered features. The final selected feature set is the one with the highest accuracy tested on the validation set, which is picked out from the training set.

### 2.5. Classification

In this study, we employ support vector machine (SVM) [44–46] with a simple linear kernel to evaluate the classification performance of our method. This technique is widely used and works well in the field of medical imaging classification [21, 30, 47]. The SVM is implemented using LIBSVM toolbox [48] with default parameters (i.e., *C* = 1). LOOCV is applied here due to limited sample size. One sample is picked out as testing sample in turn and the rest of the samples are treated as training samples. In this paper, the following three quantitative measurements are used to validate the effectiveness of our method:

(11) Accuracy=TP+TNTP+FN+TN+FP,Sensitivity=TPTP+FN,Specificity=TNTN+FP,

where TP is the number of patients correctly classified, TN is the number of healthy controls correctly classified, FP is the number of healthy controls classified as patients, and FN is the number of patients classified as healthy controls.

## 3. Results

### 3.1. Classification Performance

In this paper, to verify the effect of sparse low-rank FBN on classification performance, we conduct experiments on methods based on Pearson coefficient FBN and sparse FBN. Additionally, the methods with each single kind of features are also used for comparison, in order to evaluate the effect of combination of the eight graph-based features. Our method achieves the best classification performance compared with the contrast methods, with accuracy of 95%, sensitivity of 96.77%, and specificity of 93.10%. We can see that the results of our method are better than the methods based on Pearson coefficient FBN and sparse FBN, from Tables 1, 2, and 3. As shown in Table 1, our method performs better than the methods with any single kind of features. Besides, the results of different classifiers with sparse low-rank FBN are listed in Table 4. The parameters of all the classification methods are selected by LOOCV.

### 3.2. Effect of Regularization Parameters

**Figure 2:**
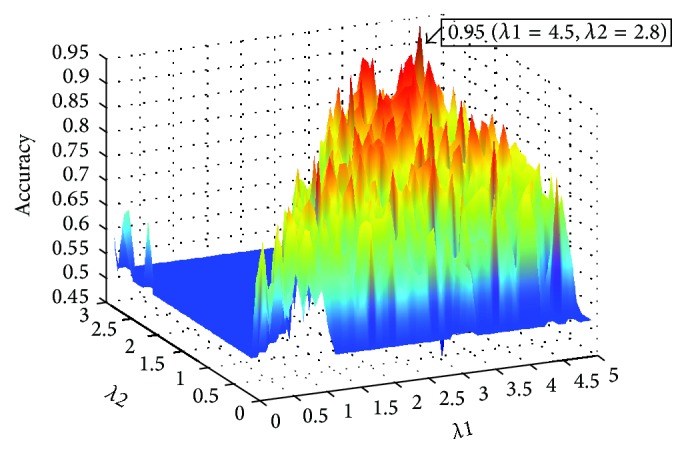
The classification accuracy with different sets of parameters.

The regularization parameters involved in the sparse low-rank model may significantly affect FBN construction and the classification performance. The optimal parameters are obtained from LOOCV. For our method, *λ*_1_ and *λ*_2_ are both in the range [0.1–5] with an increment step of 0.1. The classification accuracy of our method with different sets of parameters is shown in Figure 2. We can see that the best classification accuracy is achieved when *λ*_1_ is 4.5 and *λ*_2_ is 2.8. Therefore, this set of parameters is selected for further analysis. *λ*_1_ and *λ*_2_ are the regularization parameters for trade-off among data-fitting, sparsity constraint, and low-rank constraint. This optimal set of parameters indicates that the combination of sparsity and low-rank improves the classification performance. In addition, it can be observed that the classification performance is sensitive to the regularization parameters.

### 3.3. Analysis of Sparse Low-Rank FBN

**Figure 3:**
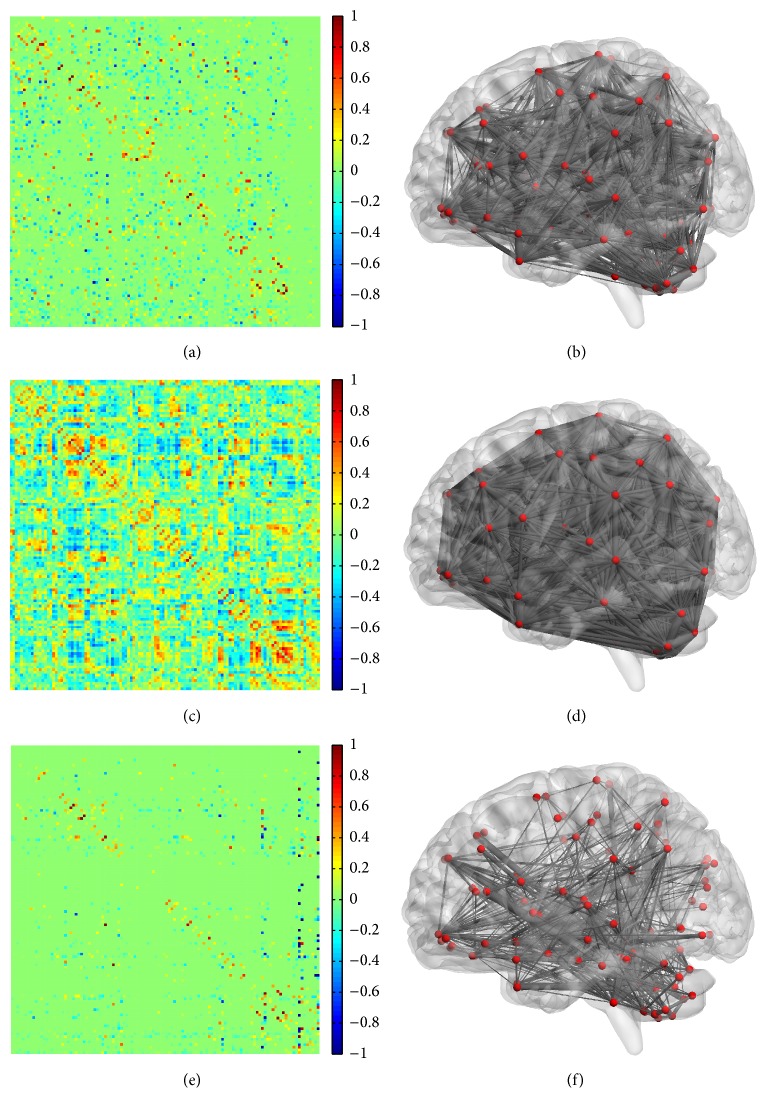
FC matrix and topology structure of FBN from one patient with depression. (a) and (b) are the FC matrix and topology structure of sparse low-rank FBN, (c) and (d) are those of Pearson correlation FBN, and (e) and (f) are those of sparse FBN.

In this paper, FBN is constructed by sparse low-rank model.  Figure 3 shows the FC matrix and topology structure of one patient with depression, which are constructed by sparse low-rank model, Pearson correlation model, and sparse representation model. The parameters used in the FBN shown in Figure 3 are optimally obtained from LOOCV. The parameters for sparse low-rank model ((a) and (b)) are 4.5 (*λ*_1_) and 2.8 (*λ*_2_). The threshold for Pearson correlation model ((c) and (d)) is 20%. The parameter for sparse representation model ((e) and (f)) is 3.2 (*λ*). It can be observed that the FC inferred by sparse representation model and sparse low-rank model can automatically remove some weak connections. Compared with sparse representation model, sparse low-rank model can lead to a clearer modular structure in the FBN. Moreover, the classification performance of methods based on sparse low-rank FBN is better than methods based on Pearson correlation FBN or sparse FBN, as mentioned in the last subsection.

**Figure 4:**
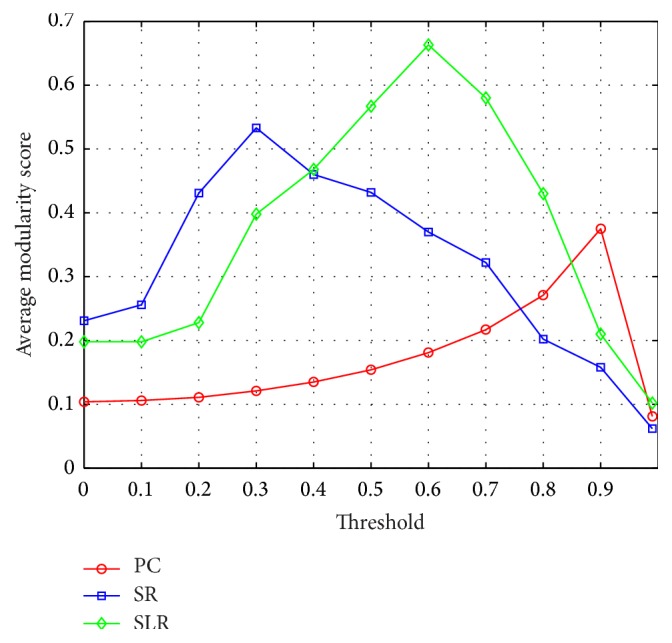
Average modularity scores of different FBN with different thresholds. PC: Pearson correlation model; SR: sparse representation model; and SLR: sparse low-rank model.

Furthermore, we use the modularity score [49] to evaluate the modularity of FBN constructed by the three models.  Figure 4 shows the average modularity scores of FBN constructed by Pearson correlation model, sparse representation model, and sparse low-rank model with different thresholds. The modularity scores shown in Figure 4 are the average modularity scores of all the subjects. Different thresholds are used in the FBN to remove weak connections in varying degrees. And the thresholds are applied to the absolute value of connections in order to obtain valid modularity scores. The connection whose absolute value is less than a certain threshold is removed. We can see from Figure 4 that sparse low-rank model can lead to a clearer modular structure in the FBN for two reasons. (1) The peak value is obtained by sparse low-rank model, compared with Pearson correlation model and sparse representation model and (2) the area under the curve of sparse low-rank model is the largest among areas of the three models. And the largest area under the curve means the maximum sum of average modularity scores with different thresholds.

### 3.4. Number of Selected Features

**Figure 5:**
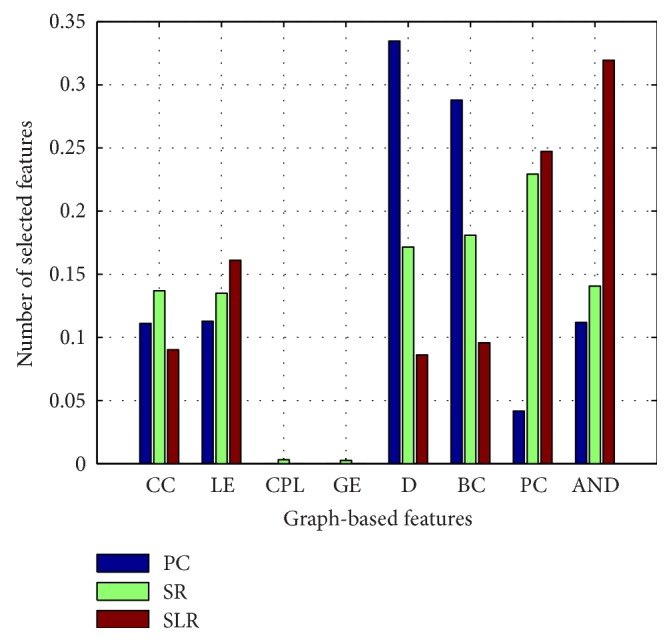
Proportion of each kind of selected features in the three methods. PC: Pearson correlation model; SR: sparse representation model; and SLR: sparse low-rank model.

After extracting the eight graph-based features, we obtain a feature vector with a size of 698 for each subject. Because of the high dimensionality of the feature vector, feature selection is essential to remove redundant features and improve the classification performance. Fisher score is used in this study to sort different dimensions of features based on the discriminatory power. We select different number of ordered features with max Fisher score to train and test the classifier. The number of selected features that resulted in the best classification performance is applied. The proportion of each kind of selected features in every LOOCV is shown in Figure 5.

### 3.5. Discriminative Brain Regions

**Figure 6:**
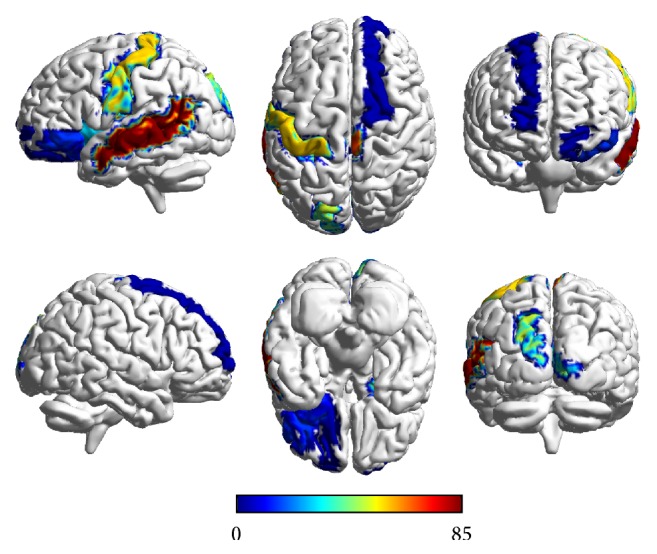
The discriminative brain regions of patients with depression compared with healthy controls. The color bar indicates the index of displayed brain regions.

The selected graph-based features are related to the specific brain regions, which contribute to the classification. These related brain regions are treated as discriminative brain regions of patients with depression compared with healthy controls, as shown in Figure 6. Specifically, we first use Fisher score to sort all the 698 dimensions of graph-based features in each LOOCV. Secondly, we use different sets with increased number of sorted features to train and test the classifier. And the number of features which results in the best performance is picked out. The selected features from the 116*∗*6 local measures are related to the specific brain regions. Finally, we count the times that each related brain region is selected. In addition, there are 12 brain regions which are picked out in all the LOOCV. The name of these brain regions and the number of times they are picked out are listed in Table 5. The discriminative brain regions include postcentral gyrus, paracentral lobule, posterior cingulate cortex, calcarine, orbital superior frontal gyrus, superior frontal gyrus, Heschl gyrus, superior occipital gyrus, amygdala, middle temporal gyrus, orbital inferior frontal gyrus, and insula.

## 4. Discussion

In this study, the proposed method, using sparse low-rank model and graph-based features, provides promising result for depression disorder classification. As shown in Table 1, our proposed method achieves the best classification performance, compared with using any single graph-based feature based on sparse low-rank FBN. We can see from Tables 1, 2, and 3 that our method performs better than Pearson correlation FBN and sparse FBN. In addition, the algorithm combining all the graph-based features outperforms the one with only one feature. Table 4 shows that linear SVM used in our method is superior to other commonly used classifiers. The highest accuracy of our method demonstrates the capability of accurately discriminating patients with depression from healthy controls. Significant improvement in sensitivity indicates the superiority of the proposed method in identifying patients with depression based on fMRI data. It is very important because misclassifying a patient to healthy control may cause severe consequences such as delaying critical treatment period.

The FBN is constructed by sparse low-rank model, which can automatically remove the weak connections and retain the modular structure. As illustrated in Figure 3, sparse low-rank model obtains sparser connection matrix than Pearson correlation model. However, the great sparsity of sparse low-rank FBN does not affect the classification performance as shown in Tables 1 and 3. On the contrary, the reserved strong connections of sparse low-rank FBN can achieve higher classification performance. Compared with sparse representation model, sparse low-rank model can capture improved modular structure as shown in Figures 3 and 4, which has been verified as an inherent property of FBN.

After constructing the FBN, we extract eight graph-based features to characterize the network and classify patients with depression and healthy controls. Because of the high dimensionality of extracted features, Fisher score algorithm is used to rank the features and select the feature set with best classification performance. We can see from Figure 5 that average neighbor degree is the most commonly selected feature in our method. However, degree and participation coefficient are the most commonly selected features in the method based on Pearson correlation FBN and sparse FBN, respectively. This finding suggests that the kind of the most effective feature is different for different methods. This is why we consider a variety of graph-based features.

The brain regions related to the selected graph-based features are the discriminative brain regions of patients with depression. As shown in Table 5, the discriminative brain regions are consistent with previous studies [4, 50], which can further prove the effectiveness of our method. Most of the discriminative brain regions are located at frontal lobe (paracentral lobule, superior frontal gyrus, orbital superior frontal gyrus, and orbital inferior frontal gyrus), occipital lobe (calcarine and orbital superior frontal gyrus), and temporal lobe (middle temporal gyrus and Heschl gyrus). The most commonly selected brain region in our method is postcentral gyrus, which is the primary somatosensory cortex [4]. Another brain region with high discrimination is posterior cingulate cortex, which has been reported as having abnormal FC in patients with depression [51]. Previous studies have indicated that posterior cingulate cortex is important for successful retrieval of self-relevant information [52]. Heschl gyrus is a primary auditory cortex and a subregion of superior temporal gyrus, which plays a key role in emotional processing and social cognition [53, 54]. It has been reported that insula is associated with abnormal interoception and pain processing in patients with depression [55]. In addition, amygdala, an important area for processing threat and orchestrating a complex set of emotional and physiologic responses [56], is also detected as discriminative brain region of depression in our study. These discriminative brain regions may help us better understand the pathogenesis of depression disorder.

## 5. Conclusion

In this paper, we develop a new method to classify fMRI data of patients with depression and healthy controls. More specifically, in order to calculate the relationship between brain regions given all the other brain regions, we first construct FBN with sparse low-rank model instead of the conventional Pearson correlation model. Our motivation also lies in that sparse low-rank model can describe the sparse and modular structure of FBN. Secondly, we extract eight graph-based features to effectively characterize the FBN from different aspects. Thirdly, Fisher score is used to rank features and select the optimal feature subset. Finally, the selected features are input to SVM for depression disorder classification. Experimental results demonstrate that our proposed method yields improved classification performance compared with the conventional methods based on Pearson correlation FBN and sparse FBN. In addition, the combination of graph-based features in our method further improves the classification performance. The promising classification result indicates our method can be used as an automatic tool to assist in diagnosis of depression disorder.

## Figures and Tables

**Table 1:** Classification performance of our method (sparse low-rank FBN).

Feature	NSF	Accuracy (%)	Sensitivity (%)	Specificity (%)
CC	8	83.33	80.65	86.21
LE	46	85.00	87.10	82.76
CPL	—	60.00	70.97	48.28
GE	—	60.00	70.97	48.28
D	22	83.33	80.65	86.21
BC	22	85.00	80.65	89.66
PC	20	83.33	83.87	82.76
AND	18	91.67	90.32	93.10
Eight features	12	95.00	96.77	93.10

NSF: number of selected features; CC: clustering coefficient; LE: local efficiency; CPL: characteristic path length; GE: global efficiency; D: degree; BC: betweenness centrality; PC: participation coefficient; and AND: average neighbor degree.

**Table 2:** Classification performance of sparse FBN.

Feature	NSF	Accuracy (%)	Sensitivity (%)	Specificity (%)
CC	10	81.67	80.65	82.76
LE	10	83.33	77.42	**89**.**66**
CPL	—	55.00	45.16	65.52
GE	—	53.33	48.39	58.62
D	54	83.33	80.65	86.21
BC	113	73.33	77.42	68.97
PC	6	73.33	61.29	86.21
AND	10	68.33	74.19	62.07
Eight features	70	**85**.**00**	**83**.**87**	86.21

**Table 3:** Classification performance of Pearson correlation FBN.

Feature	NSF	Accuracy (%)	Sensitivity (%)	Specificity (%)
CC	17	73.33	70.97	75.86
LE	23	78.33	77.42	79.31
CPL	—	55.00	58.06	51.72
GE	—	56.67	51.61	62.07
D	46	78.33	77.42	79.31
BC	8	78.33	83.87	72.41
PC	86	81.67	83.87	79.31
AND	1	70.00	61.29	79.31
Eight features	65	83.33	83.87	82.76

**Table 4:** Classification performance of the most commonly used classifiers.

Classifier	NSF	Accuracy (%)	Sensitivity (%)	Specificity (%)
NB	15	88.33	87.10	89.66
*k*-NN	17	88.33	90.32	86.21
LDA	11	90.00	90.32	89.66
SVM (RBF)	11	90.00	93.55	86.21
SVM (linear)	12	95.00	96.77	93.10

NB: naive Bayes; *k*-NN: *k*-nearest neighbors; and LDA: linear discriminant analysis.

**Table 5:** The discriminative brain regions of patients with depression and the number of times that they are picked out.

Brain regions	NTPO	Related studies
Postcentral_L	151	Guo et al. [4]
Paracentral_Lobule_R	121	Kenny et al. [50]
Cingulum_Post_R	118	Zhu et al. [51]
Calcarine_R	96	Zhang et al. [57]
Frontal_Sup_Orb_L	62	Drevets et al. [58]
Frontal_Sup_R	60	Zhang et al. [57]
Heschl_L	58	Amico et al. [53]
Occipital_Sup_L	49	Zhang et al. [6]
Amygdala_R	2	Zhang et al. [6]
Temporal_Mid_L	1	Zhang et al. [57]
Frontal_Inf_Orb_L	1	Drevets et al. [58]
Insula_L	1	Liu et al. [3]

NTPO: number of times that they are picked out.
